# CLASP1 regulates endothelial cell branching morphology and directed migration

**DOI:** 10.1242/bio.028571

**Published:** 2017-08-31

**Authors:** Nicole M. Myer, Kenneth A. Myers

**Affiliations:** Department of Biological Sciences, University of the Sciences in Philadelphia, Philadelphia PA 19104, USA

**Keywords:** CLASP, Endothelial cell, Extracellular matrix, Microtubule, Migration, Polarity

## Abstract

Endothelial cell (EC) branching is critically dependent upon the dynamic nature of the microtubule (MT) cytoskeleton. Extracellular matrix (ECM) mechanosensing is a prominent mechanism by which cytoskeletal reorganization is achieved; yet how ECM-induced signaling is able to target cytoskeletal reorganization intracellularly to facilitate productive EC branching morphogenesis is not known. Here, we tested the hypothesis that the composition and density of the ECM drive the regulation of MT growth dynamics in ECs by targeting the MT stabilizing protein, cytoplasmic linker associated protein 1 (CLASP1). High-resolution fluorescent microscopy coupled with computational image analysis reveal that CLASP1 promotes slow MT growth on glass ECMs and promotes short-lived MT growth on high-density collagen-I and fibronectin ECMs. Within EC branches, engagement of either high-density collagen-I or high-density fibronectin ECMs results in reduced MT growth speeds, while CLASP1-dependent effects on MT dynamics promotes elevated numbers of short, branched protrusions that guide persistent and directed EC migration.

## Abstract (precis)

**Summary:** CLASP1 modulates microtubule dynamics with sub-cellular specificity in response to extracellular matrix density and composition. CLASP1 effects on microtubules promote short, branched protrusions that guide persistent and directional EC migration. This article has an associated First Person interview with the first author of the paper as part of the supplementary information.

## INTRODUCTION

Endothelial cell (EC) branching is critically dependent upon the dynamic nature of the microtubule (MT) cytoskeleton. MT dynamics are controlled by families of proteins that interact with and modify the stability of the MT lattice, termed MT-associated proteins (MAPs) (Wantanabe et al., 2005; Akhmanova and Steinmetz, 2010; Kumar and Wittmann, 2012). Cell signaling cascades are known to spatially and temporally target specific MAPs, and this results in the regulation of MT dynamics in one part of the cell that is distinct from MT regulatory events in another part of the cell, ultimately driving changes in cell morphology. These changes in morphology are necessary for cells to branch, establish polarity, and to establish physical contacts with the extracellular matrix (ECM) as well as to interact with other cells (Grinnell, 2000; Davis and Bayless, 2003; Stehbens and Wittmann, 2012; Wolf et al., 2013). In migrating fibroblasts, for example, MT growth establishes a leading edge defined by a protruding lamellapodia that drives the cell front forward, while MT disassembly in the cell rear promotes acto-myosin-mediated disengagement of the trailing edge of the cell (Gerhardt and Betsholtz, 2005; Kumar et al., 2012). In this way, the regulation of MT dynamics provides an essential cellular mechanism for developing a polarized morphology necessary for persistent directional migration. Additionally, MT growth dynamics (i.e. growth speeds and lifetimes) are known to be locally regulated in response to ECM compliance (Lo et al., 2000; Myers et al., 2011), while cell branching is known to be promoted by ECM compliance and 3D ECM engagement (Discher et al., 2005; Fischer et al., 2009; Grinnell and Petroll, 2010; Rehfeldt et al., 2012). Despite these findings, it remains unclear how physical signaling cues that are linked to cell engagement of the ECM result in the regulation of MT dynamics inside the cell. Additionally, it is unclear how MT dynamics are targeted for regulation in response to ECM composition and density, and the effects on EC branching and directional migration.

Cytoplasmic linker associated protein 1 (CLASP1) is a MAP that belongs to the +Tip family of proteins. It promotes stabilization of the MT lattice against catastrophe and also functions as a MT growth-promoting factor (Akhmanova and Steinmetz, 2008). For example, MTs have been shown to undergo depolymerization only until they reach a site on the MT lattice where CLASP1 molecules are present in high concentrations (Al-Bassam et al., 2010). In this way, CLASP1 plays a unique role in monitoring and modifying MT stability, by inhibiting MT disassembly and simultaneously promoting MT growth (Akhmanova et al., 2001; Galjart, 2005; Mimori-Kiyosue et al., 2005; Al-Bassam et al., 2010). CLASP1 was shown to preferentially bind to leading edge MTs (Akhmanova et al., 2001; Wittmann et al., 2003; Galjart, 2005) and to colocalize with MT End Binding (EB) protein – a ubiquitous +Tip that autonomously tracks with growing MTs and associates with other +Tips to target them to the plus end (Mimori-Kiyosue, 2005; Slep et al., 2005; Matov et al., 2010). Upon siRNA depletion of CLASP1, EB proteins displayed a partial redistribution along the MT lattice, though there was still significant localization to the MT plus end (Grimaldi et al., 2014). Affinity of CLASP1 for EB proteins and the MT lattice is negatively modulated by phosphorylation events triggered upstream by the small GTPase Rac1 and its effector protein, GSK3β (Wittmann and Waterman-Storer, 2005; Kumar et al., 2012). Pathways that enhance podosome formation also stimulate CLASP-MT binding, facilitating MT polymerization (Efimova et al., 2014). However, the contribution of CLASP1-mediated regulation of MT growth dynamics to the formation of EC branched protrusions or to EC directional migration has not yet been investigated.

It is well established that in addition to regulation via MAPs, MT dynamics are also regulated in response to the physical nature of their environment (Adams and Watt, 1993; Wang et al., 1993; Waterman-Storer and Salmon, 1997; Putnam et al., 1998; Ingber, 2002; Polte, 2004; Mammoto et al., 2009). Quantifiable ECM factors, like composition, density, and tensile force potential, also contribute to the regulation of cell morphogenesis and motility (Wang et al., 1993; Putnam et al., 2000; Hsu et al., 2010; Parsons et al., 2010). For example, regulation of cell signaling during angiogenesis and maintenance of the structural integrity of blood vasculature have been shown to depend on the protein composition of the ECM, including collagen and fibronectin (Davis et al., 2000, 2002; Davis and Bayless, 2003; Wolf and Friedl, 2011). Changes in ECM density have been previously shown to alter cell shape via creation of an alternative cell-ECM force balance (Mooney et al., 1995), while elevated fibronectin and collagen-I densities are known to influence the level of polymerized MTs and to promote increased MT mass (Putnam et al., 2000). In addition, a variety of adherent cell types have been shown to modify the expression of specific integrins in order to promote physical engagement of the ECM dependent upon composition (Davis and Camarillo, 1995; Kim et al., 2000; Senger et al., 2002; Smith et al., 2010). Additionally, CLASP-mediated MT tethering at focal adhesions has been shown to modify the ECM by promoting the delivery of vesicles containing matrix metalloproteinases, suggesting that CLASP is important in ECM degradation and focal adhesion turnover (Stehbens et al., 2014). These data support a model in which CLASPs play a central role in coupling physical ECM signaling cues to the modulation of MT growth dynamics; yet it remains unknown how ECM composition and density influence CLASP1-mediated control of MT dynamics.

To better understand the combined influence of CLASP1 and physical ECM interactions on MT dynamics and migration during EC migration, we sought to determine if MT dynamics were regulated based on differences in ECM composition and/or ECM concentration in human umbilical vein endothelial cells (HUVECs; hereafter referred to as ECs). To accomplish this, we experimentally modified CLASP1 expression levels and performed live-cell imaging followed by computational image analysis of MT growth dynamics on low- or high-density type-1 collagen or fibronectin ECMs. Cumulatively our results indicate that CLASP1 promotes slow and short-lived MT dynamics within the cell body, while CLASP1 promotes slow MT growth within EC branches. Additionally, we identify that high-density ECM engagement modulates MT growth dynamics through a CLASP1 signaling pathway in order to promote EC branching and directional migration.

## RESULTS

### CLASP1 promotes slow MT assembly

**Fig. 1.**
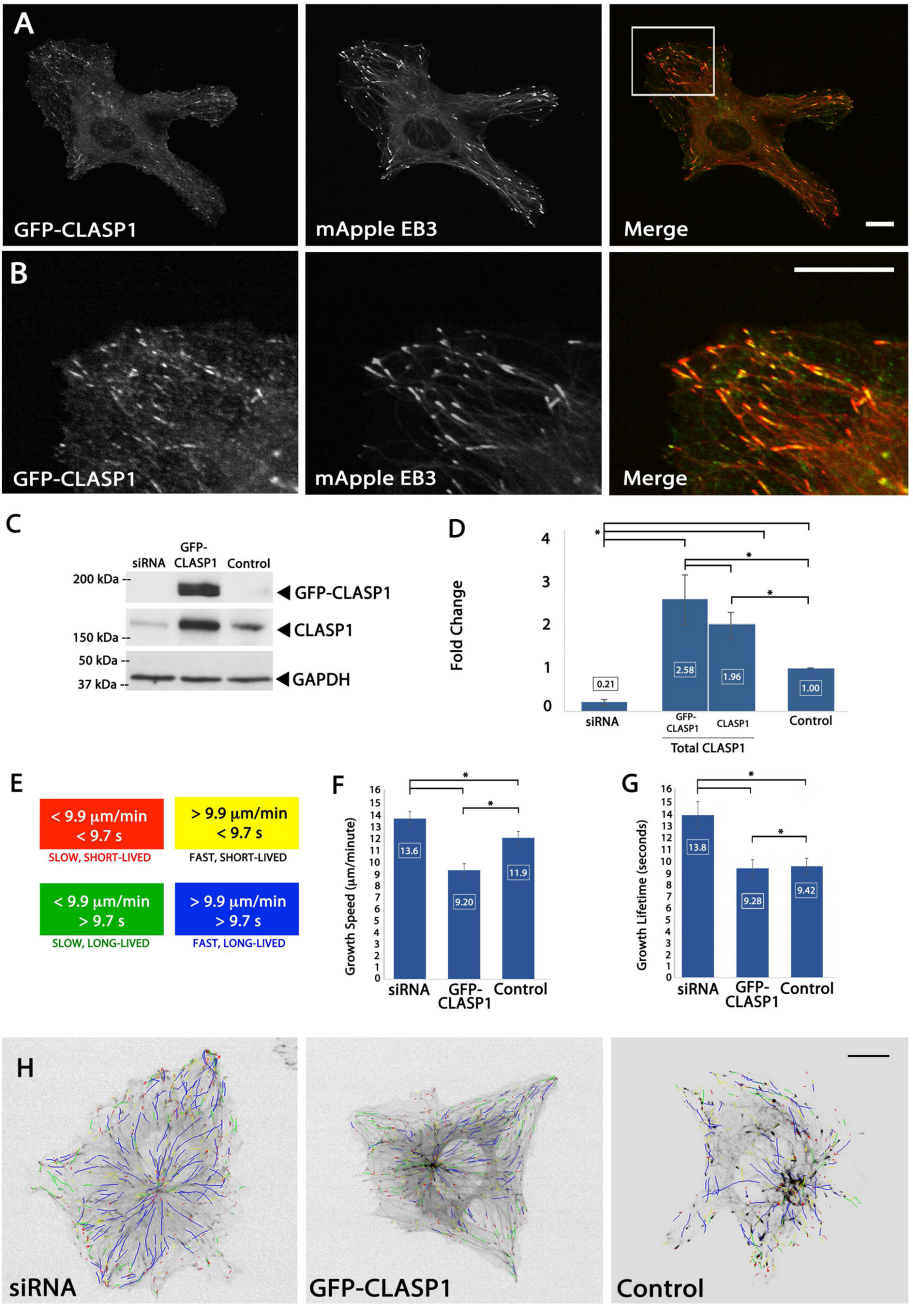
**CLASP1 promotes slow MT assembly.** (A) Fluorescent images of an EC expressing GFP-CLASP1 (left panel) and mApple EB3 (middle panel) and merge (right panel). (B) Zoomed (4×) images from white-boxed area in respective images from A. Merged image demonstrates co-localization at the MT plus-end. (C) Western blot of whole-cell lysate of ECs. (D) Average densitometry of three individual western blots quantifying knockdown, overexpression, and endogenous CLASP1 (control). (E) Color scheme for the four subpopulations of MT growth tracks depicted in H (*n* cells=336; *n* tracks=79,469). (F,G) Bar graph depicting the mean MT growth speeds (F) and mean MT growth lifetimes (G). (H) plusTipTracker generated MT growth track overlays from 2 min time-lapse videos of mApple-EB3 (images acquired at 2 s intervals). Scale bars in A, B and H: 10 µm. D: *P<0.001; F,G: *P<0.05; ANOVA. Error bars indicate ±s.e.m.

We first set out to confirm that GFP-CLASP1 tracks with polymerizing MT plus ends in ECs, similar to what has previously been described in other cell lines (Akhmanova et al., 2001; Mimori-Kiyosue, 2005; Wittmann and Waterman-Storer, 2005). Time-lapse image analysis of live ECs co-expressing a fluorescently-labeled CLASP1 protein (GFP-CLASP1) and fluorescently-labeled MT EB protein 3 (mApple-EB3) revealed that GFP-CLASP1 tracked with mCherry-EB3 on growing MT plus ends in ECs (Fig. 1A,B).

Using western blot densitometry, we first established that siRNA reduced levels of CLASP1 by an average of 79%, and that overexpression of GFP-CLASP1 enhanced CLASP1 levels by 258% (Fig. 1C,D). We next sought to determine the effects of CLASP1 on MT dynamics in ECs cultured on glass coverslips in the absence of a biological ECM substrate. Live-cell, time-lapse imaging was performed on ECs expressing mApple-EB3 together with either CLASP1-siRNA targeted to the 3′ UTR of CLASP1 (siRNA), GFP-CLASP1, or a non-target siRNA (control).

To assess the influence of CLASP1-mediated regulation of MT dynamics, we measured MT growth speed and growth lifetime in cells with altered CLASP1 expression levels using plusTipTracker automated image analysis of time-lapse images of mApple-EB3 (Applegate et al., 2011; Myers et al., 2011; D'Angelo et al., 2017). Comparison of MT growth parameters revealed that CLASP1 siRNA resulted in faster MT growth speeds, whereas overexpression of GFP-CLASP1 resulted in slower MT growth speeds (Fig. 1F). Interestingly, while siRNA depletion of CLASP1 resulted in longer-lived MT growth, GFP-CLASP overexpression resulted in MT growth lifetimes that were statistically similar to controls, suggesting that endogenous levels of CLASP1 protein may be sufficient to regulate MT growth lifetimes in ECs cultured on a glass ECM (Fig. 1G). Qualitative analysis of image overlays of MT plus-end motion tracks showed that depletion of CLASP1 induced fast and long-lived MT growth (blue tracks), while GFP-CLASP1 overexpression induced slow and short-lived MT growth (red tracks) throughout the cell (Fig. 1E,H). Together these results indicate that CLASP1 tracks with growing MT plus ends and promotes slow MT growth speeds in ECs cultured on a glass ECM.

### ECM density regulates MT growth lifetime in a CLASP1-dependent manner

Cytoskeletal components have been shown to generate forces that are transmitted to the ECM during branching morphogenesis and migration (Wittmann and Waterman-Storer, 2001; Stehbens and Wittmann, 2014; Thievessen et al., 2015). Additionally, ECM mechanosensing has been shown to regulate cell morphology and migration through mechanisms that target the regulation of the MT cytoskeleton (Fischer et al., 2009; Myers et al., 2011; D'Angelo et al., 2017). Collagen-I and fibronectin are physiological components of the ECM that are known to elicit signaling cues to ECs during physical cell-ECM engagement (Jin et al., 2011; Plotnikov et al., 2012; Digiacomo et al., 2016; Jiang et al., 2016). These signaling cues are essential for promoting vascular development and for maintaining vessel structural integrity (Rhodes and Simons, 2007; Kusuma et al., 2012). We set out to determine whether ECM concentration (henceforth termed ECM ‘density’) contributes to CLASP1-mediated regulation of MT growth dynamics. ECs were cultured on high (90 µg/ml) or low (45 µg/ml) collagen-I densities and on high (100 µg/ml) or low (10 µg/ml) fibronectin densities. These concentrations were chosen based upon the range of collagen-I and fibronectin densities used in previous studies of MT growth dynamics and to maintain the concentration of collagen-I below the critical concentration for collagen-I polymerization (approx. 100 µg/ml) (Fischer et al., 2009; Kanchanawong et al., 2010; Myers et al., 2011; Chen et al., 2013; Braun et al., 2014; Burnette et al., 2014; Grimaldi et al., 2014).

**Fig. 2.**
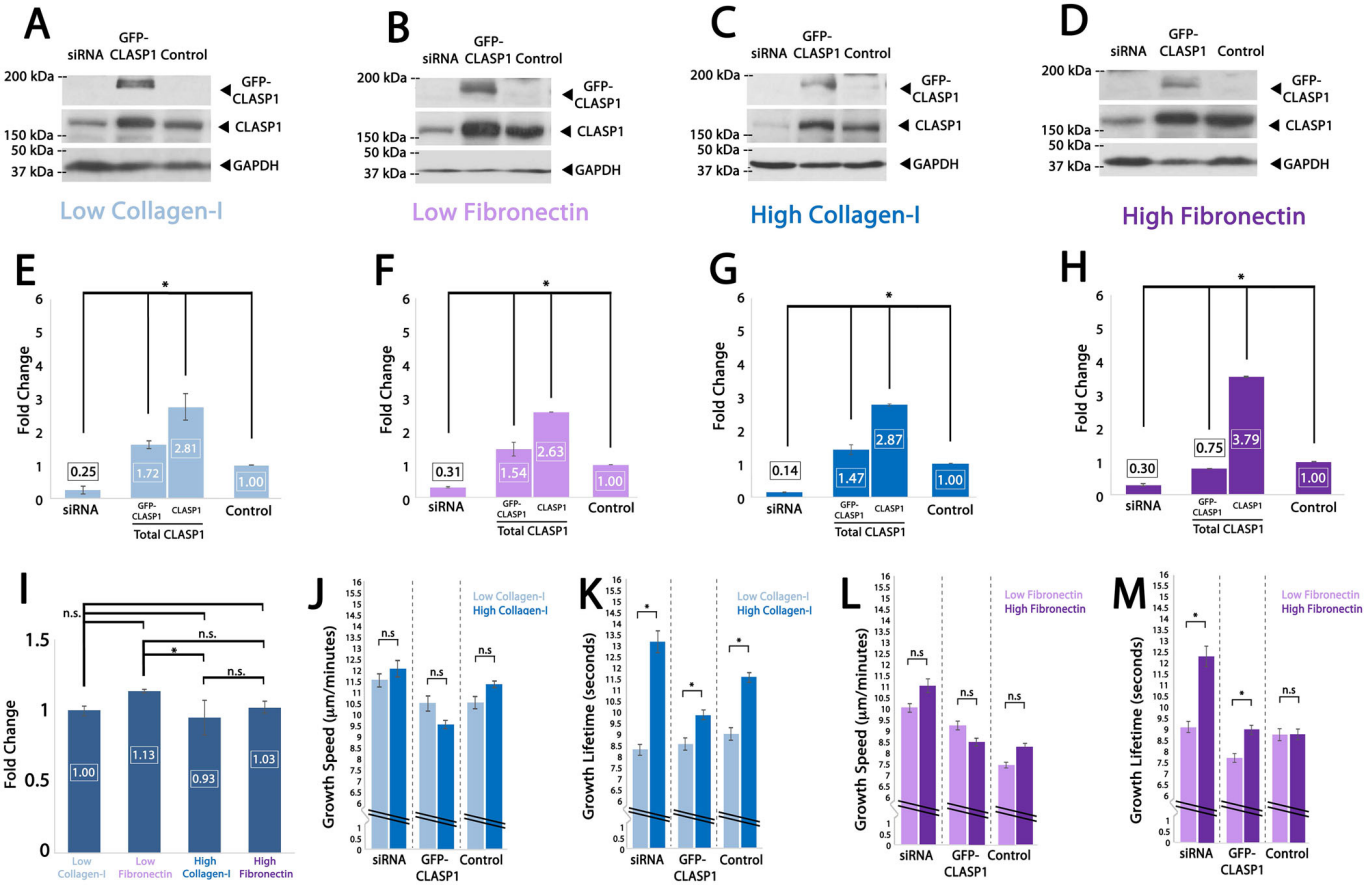
**ECM density regulates MT growth lifetimes in a CLASP1-dependent manner.** (A-D) Western blot of whole-cell lysate of ECs on low- or high-density collagen-I or low- or high-density fibronectin. (E-H) Average densitometry of three individual western blots quantifying knockdown, overexpression, and endogenous CLASP1 (control). (I) Comparison of control densitometry measurements from each of the above mentioned ECMs. CLASP1 expression levels were normalized to those measured in ECS cultured on low collagen I ECMs. (J,K) Comparison of mean MT growth speeds (J) and growth lifetimes (K). Light blue bars denote low-density collagen-I (45 µg/ml) and dark blue bars denote high-density collagen-I (90 µg/ml). (L,M) Comparison of mean MT growth speeds (L) and growth lifetimes (M). Light purple bars denote low-density fibronectin (10 µg/ml) and dark purple bars denote high-density fibronectin (100 µg/ml). E-I: *P<0.001; J-M: *P<0.05; n.s., not statistically different; ANOVA. Error bars indicate ±s.e.m.

We first set out to determine the effects of ECM composition (collagen-I versus fibronectin) and density (low versus high) on endogenous CLASP1 protein levels and CLASP1 protein levels under conditions of experimental manipulation. Western blot densitometry analysis revealed that on low collagen-I ECMs and low fibronectin ECMs, total CLASP1 protein (endogenous CLASP1 plus GFP-CLASP1) levels were elevated to 453% and 417% of control, respectively; while CLASP1 siRNA was effective in lowering CLASP1 protein levels 25% and 31% of control values on low-collagen-I and low-fibronectin ECMs, respectively (Fig. 2A,B,E,F). Comparing high-collagen-I ECMs and high-fibronectin ECMs, total CLASP1 protein levels were elevated to 434% and 454% of control, respectively; while CLASP1 siRNA was effective in lowering CLASP1 protein levels to 14% and 30% of control values on low-collagen-I and low-fibronectin ECMs, respectively (Fig. 2C,D,G,H). Taken together, these data suggest that CLASP1 siRNA knockdown and GFP-CLASP1 overexpression results in similar relative CLASP1 protein levels independent of ECM substrate composition or density. Additionally, we used western blot densitometry to evaluate the effects of ECM substrate composition and substrate density on endogenous CLASP1 protein levels. This analysis revealed similar endogenous levels of CLASP1 on all ECM substrates except that CLASP1 was slightly but significantly elevated on low fibronectin compared to high collagen (Fig. 2I). To account for this variation, we excluded comparisons of MT growth dynamics between these two ECM substrates.

We next set out to determine the effects of ECM composition and density on MT growth dynamics. Comparing ECs cultured on low- versus high-density collagen-I ECMs, MT growth lifetimes were increased on high-density collagen-I ECMs, but there was no collagen-I density-dependent change measured in MT growth speeds (Fig. 2J,K). Experimental manipulation of CLASP1 revealed statistically significant effects on MT growth lifetimes that were observed only on high-collagen-I ECMs. Compared to control conditions, CLASP1 siRNA induced significantly longer-lived MT growth, while GFP-CLASP1 overexpression resulted in the shortest-lived MT growth events measured on high-density collagen I ECMs. These results suggest that type-I collagen density regulates MT growth lifetimes in a CLASP1-dependent manner.

Comparison of high- versus low-density fibronectin ECMs revealed that, unlike on collagen ECMs, MT dynamics were unresponsive to changes in fibronectin density alone (Fig. 2L,M; Control). However, CLASP1 siRNA resulted in significantly longer-lived MT growth lifetimes only on high-density fibronectin ECMs, while GFP-CLASP1 growth lifetimes remained similar to control (Fig. 2M). These results suggest that CLASP1 is required to inhibit MT growth lifetimes on high-density fibronectin ECMs.

In addition to changes in the dynamics of MT assembly influenced by ECM engagement and CLASP1, we also identified that the number of MT assembly events (‘track no.’) was modulated both by CLASP1 and ECM condition. Analysis of MT growth track number revealed that CLASP1 siRNA resulted in a reduction in MT growth track number on all ECMs except for low-density collagen ECMs, where CLASP1 siRNA had the highest MT growth track number (Table 1). Measured changes in MT track number show that CLASP1 knockdown contributes to the regulation of the number of MT growth events in response to ECM density, which is likely to influence the overall organization of the EC MT array. Further, these data highlight that changes in MT growth track number occur independently of the effects of ECM density or CLASP1 manipulation on MT growth speed and MT growth lifetime.

**Table 1.**
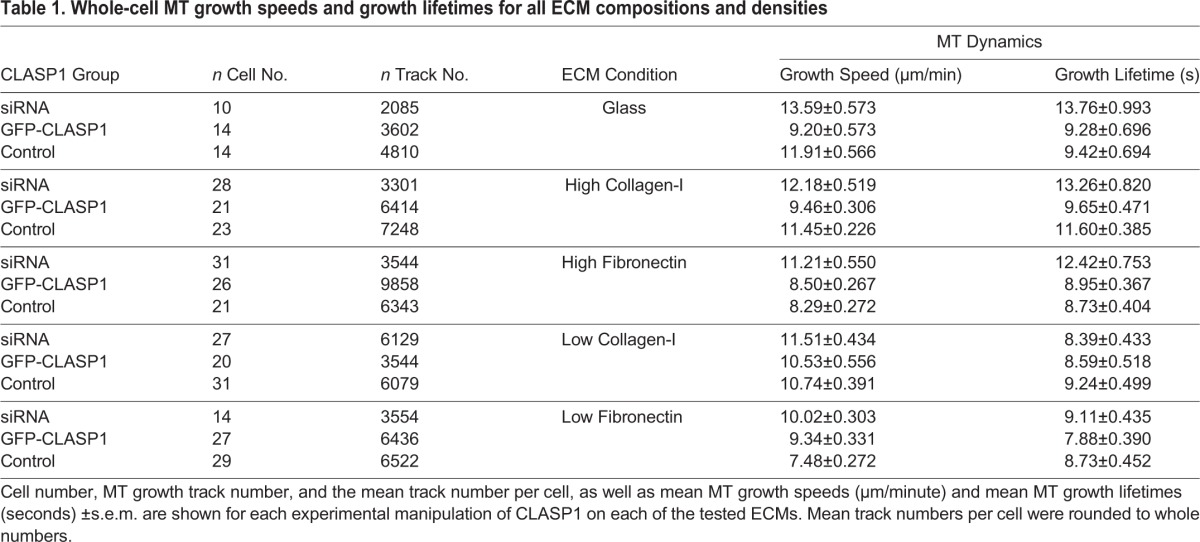
**Whole-cell MT growth speeds and growth lifetimes for all ECM compositions and densities**

### CLASP1 regulates MT growth dynamics and EC branching morphology in response to ECM composition

A combination of both soluble and physical signaling cues are known to drive EC branching morphogenesis and migration through differential regulation of MT growth dynamics (Eilken and Adams, 2010; Ma and Waxman, 2008; Myers et al., 2011; Braun et al., 2014). Mechanistic control of CLASP1 through spatiotemporal inhibition/activation of small GTPases has been shown to play a critical role in regulating MT dynamics (Akhmanova et al., 2001; Wittmann and Waterman-Storer, 2005). ECs are known to exhibit differential morphologies in response to variations in ECM composition (Schlie-Wolter et al., 2013), and the control of EC shape, such as branching morphology, is known to rely on localized regulation of MT growth dynamics in order to guide EC migration (Myers et al., 2011). We therefore set out to determine the effects of ECM composition on CLASP1-mediated regulation of MT dynamics. To do this we compared control ECs to CLASP1-siRNA knockdown and GFP-CLASP1 overexpression that were cultured on high-density collagen-I ECMs, and compared them to high-density fibronectin ECMs. We focused our investigation on high-density collagen-I and high-density fibronectin ECMs because analysis of MT growth speeds and growth lifetimes were significantly altered only on high-density ECMs (see Fig. 2J-M).

**Fig. 3.**
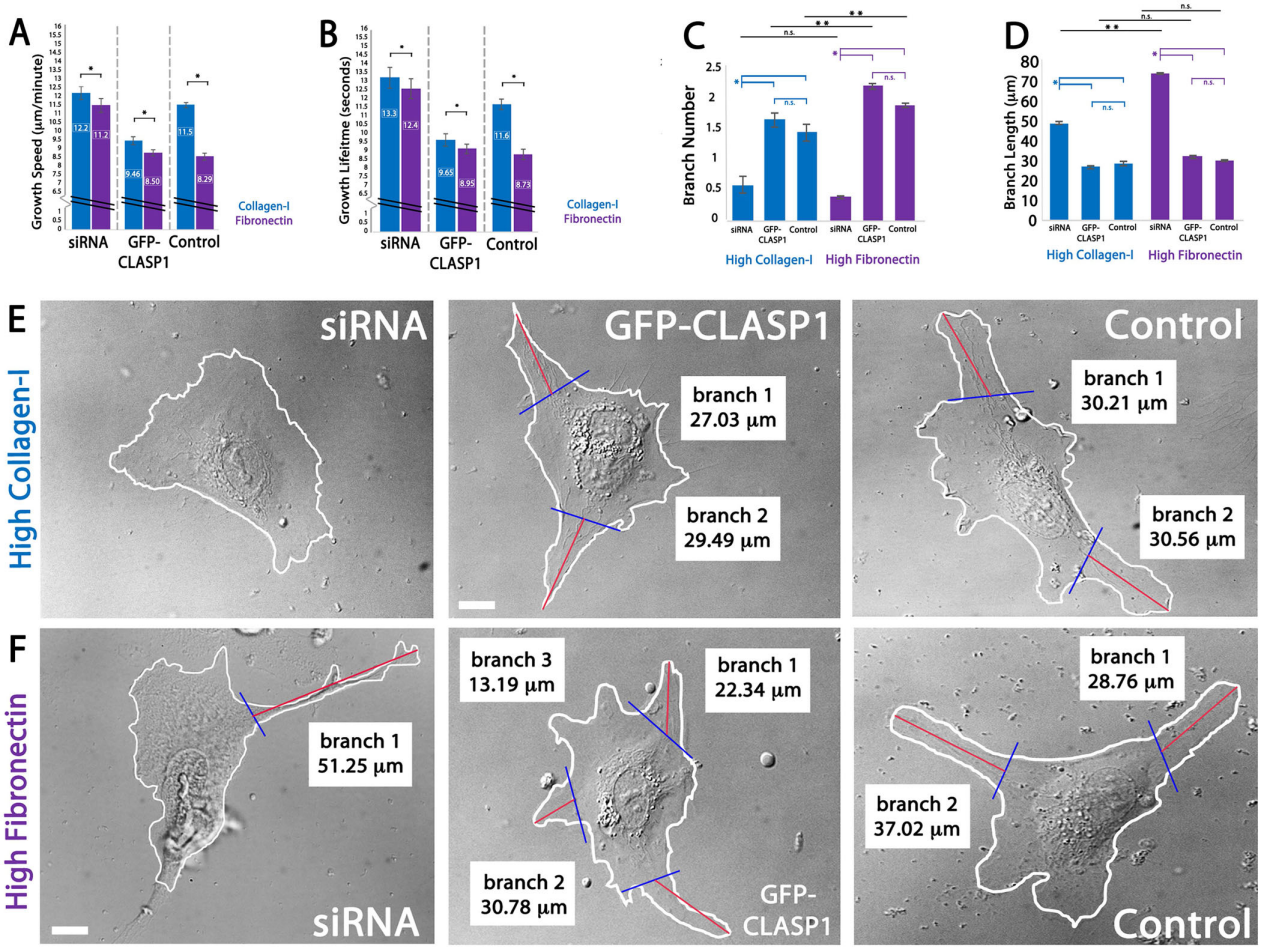
**CLASP1 regulates MT growth dynamics and EC branching morphology in response to ECM composition.** (A,B) Comparison of mean MT growth speeds (A) and mean MT growth lifetimes (B) on either high-density collagen-I (blue) or high-density fibronectin (purple) ECMs. (C,D) Comparison of mean EC branch number (C) and mean branch length (D). (E,F) EC branching when cultured on high-density collagen-I (E) or high-density fibronectin ECMs (F). Blue lines represent branch origin and red lines represent branch length. Measurements of Individual branch and branch length are displayed for each representative cell. Scale bars: 10 µm. A,B: *P<0.05; C,D: *P<0.001; ANOVA. Error bars indicate ±s.e.m.

Analysis of MT growth parameters revealed that fibronectin promoted slower and shorter-lived MT dynamics, as compared to collagen-I (Fig. 3A,B; Movies 1-6). In all cases, MT growth speeds and growth lifetimes were the fastest and longest-lived in ECs cultured on collagen-I ECMs. This difference was the most dramatic in control ECs, where MT growth speeds on collagen-I were 72.1% faster and 75.3% longer-lived than control ECs cultured on fibronectin. siRNA knockdown of CLASP1 resulted in a further increase in MT growth speed and growth lifetimes on both ECMs, but caused the most significant increase in ECs cultured on fibronectin ECMs (growth speed increase=75.9%; growth lifetime increase=72.3%). These data suggest that CLASP1 knockdown results in a loss of sensitivity to ECM composition-mediated regulation of MT dynamics, that high-density collagen-I ECMs promote fast and long-lived MT growth and that CLASP1 counteracts this effect by promoting slow and short-lived MT growth.

Previous investigations have shown that MT assembly and disassembly rates are a critical determinant for both the number and length of branching phenotypes in various cell types (Dent and Kalil, 2001; Dehmelt et al., 2003; Wantanabe et al., 2005; Kaverina and Straube, 2011; Wright et al., 2015). Additionally, mechanosensitive ECM-mediated regulation of MT growth dynamics has been shown to contribute to the branching morphology of ECs (Myers et al., 2011). We next set out to establish whether the measured effects of CLASP1 on MT growth dynamics were sufficient to promote differential branching morphogenesis on collagen-I and fibronectin ECMs. To measure EC branching morphology, we compared EC branch number and branch length in CLASP1 siRNA, GFP-CLASP1, and control ECs. On both high-density collagen-I and high-density fibronectin, branch number was significantly reduced in CLASP1 siRNA-transfected cells, while branches that did form in the siRNA group were longer than all other experimental groups (Fig. 3C-F). Compared to CLASP1 siRNA, GFP-CLASP1 expressing ECs displayed an increased number of short branches on both collagen-I and fibronectin ECMs. Mean branch numbers were significantly lower on collagen-I ECMs as compared to fibronectin ECMs in all conditions except for the siRNA group (Fig. 3C), while mean branches lengths were significantly longer in CLASP1 siRNA ECs cultured on fibronectin, as compared to siRNA ECs cultured on collagen-I (Fig. 3D). Taken together with MT dynamics measurements, our findings show that CLASP1 functions to suppress MT growth speeds and growth lifetimes in response to high-density collagen-I and high-density fibronectin ECMs. Further, our results suggest that CLASP1-mediated promotion of slow and short-lived MT growth results in increased EC branch number and may also function to suppress EC branch elongation.

### CLASP1 promotes slow MT assembly within EC branches

MT assembly dynamics are polarized to promote EC branching (Myers et al., 2011) and to promote directional migration in other cell types (Waterman-Storer and Salmon, 1997; Wadsworth, 1999; Wittmann et al., 2003; Rösner et al., 2007; Yoo et al., 2013; Braun et al., 2014; Sakakibara et al., 2014). Whole-cell investigations of CLASP1-mediated effects on MT growth dynamics revealed similar responses to variations in ECM density (Fig. 2J-M), while displaying the most robust responses to variations in ECM composition (Fig. 3A,B). Additionally, analysis of EC branching revealed that CLASP1 knockdown reduced branch number and significantly increased branch length (Fig. 3C-F). Based on these experimental outcomes, we next set out to test the hypothesis that MT growth dynamics are regulated by CLASP1 with regional specificity, within cell branches and within the cell body.

**Fig. 4.**
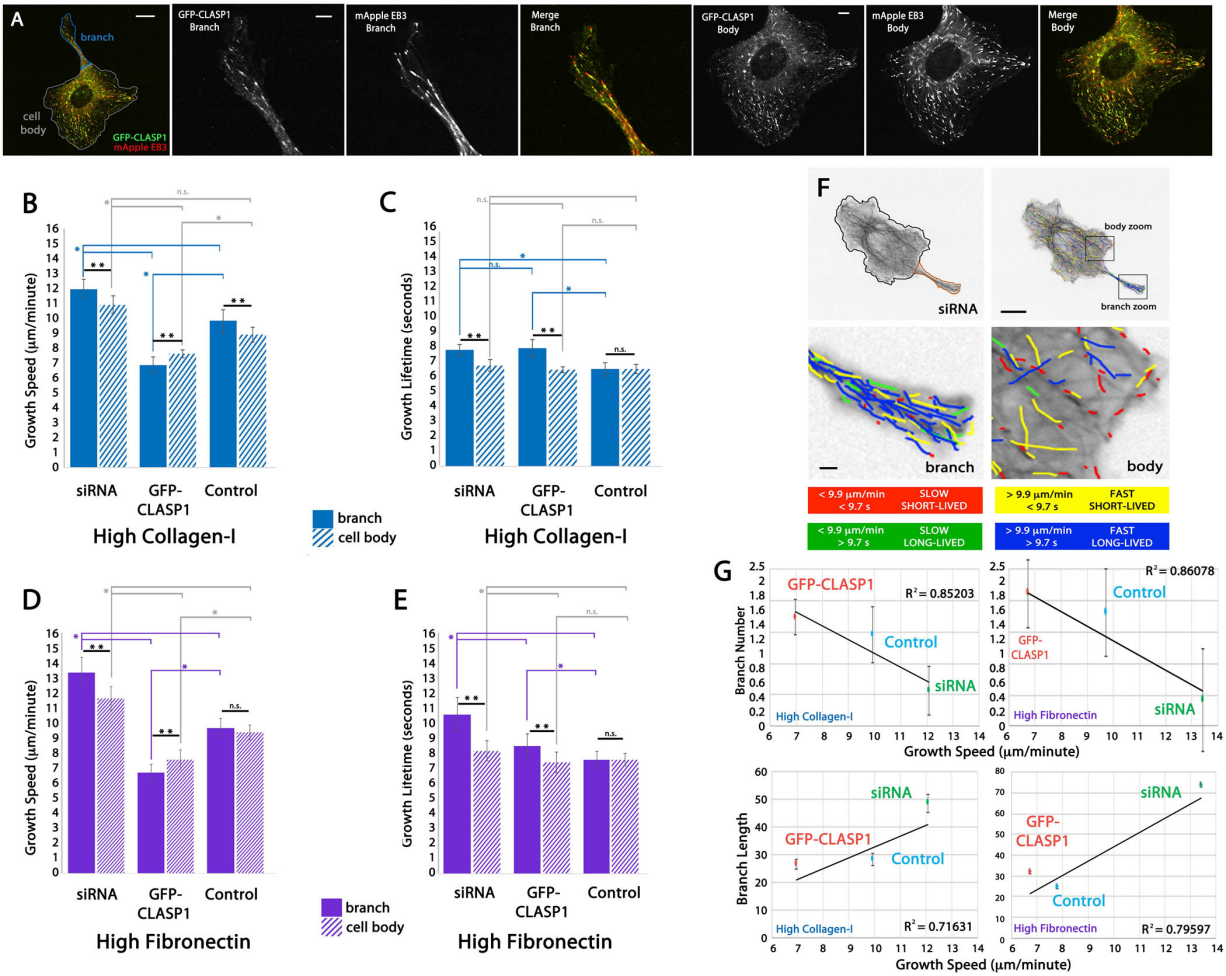
**CLASP1 promotes slow MT dynamics within EC branches.** (A) Fluorescent images of an EC expressing GFP-CLASP1 (green, far left panel and merge) and mApple EB3 (red, far left panel and merge). Merged images demonstrate co-localization of CLASP1 with EB3-labeled, growing MT plus-ends. CLASP1 displayed a uniform distribution throughout the entire cell. (B-E) Comparison of mean MT growth speeds (B and D) and mean MT growth lifetimes (C and E) in ECs cultured on high-density collagen-I (blue bars in B and C) or high-density fibronectin (purple bars in D and E). Solid bars represent dynamics within cell branches and striped bars represent dynamics within the cell body. Statistical comparisons compare branch and body means (**black significance bars), or compare branch-to-branch means (*colored significance bars) or body-to-body means (gray significance bars). (F) Still image from a time-lapse video of a CLASP1 knockdown EC expressing mApple-EB3 (top left panel). The branch is outlined in orange and the cell body is outlined in black. Overlays of MT growth tracks from the whole cell (top right panel) and zoomed regions of branch (bottom left panel) and body (bottom right panel) regions of interest are shown. MT tracks are color coded according to their growth speeds and lifetimes. (G) Regression analysis showing the correlation between branch number (top) and branch length (bottom) to average MT growth speeds within cell branches on either high-density collagen-I (left graphs) or high-density fibronectin (right graphs). Linear trend line is shown in black along with regression linearity (R^2^) values. Main scale bar: 10 µm; zoomed scale bars: 2 µm. *P<0.05; **P<0.05; n.s., not significant; ANOVA. Error bars indicate ±s.e.m.

For regional analysis of MT growth dynamics, we again chose to focus our study on ECs cultured on high-density collagen-I ECMs and high-density fibronectin ECMs, because these substrates revealed CLASP1-dependent effects on MT growth dynamics within whole cells (see Figs 2 and 3). ECs expressing mApple-EB3 together with control, CLASP1-siRNA, or GFP-CLASP1 were allowed to adhere and form branches without bias. Subsequent microscopic examination of GFP-CLASP1 and mApple EB3 within cell branches and within the cell body revealed that CLASP1 co-localized with EB3 without regional bias (Fig. 4A). Comparison of MT growth dynamics within cell branches were compared to the cell body and revealed that, in control ECs, MT growth speeds were increased within branches only on high-density collagen-I ECMs, while MT growth lifetimes were similar within the cell branches and cell bodies, independent of ECM composition (Fig. 4B-E). These findings suggest that regional regulation of MT growth speed by endogenous CLASP1 is sensitive to high-density collagen-I ECMs.

Regional analysis of MT dynamics in ECs expressing GFP-CLASP1 revealed that MT growth speeds were reduced within cell branches (Fig. 4B,D) and MT growth lifetimes were increased within cell branches (Fig. 4C,E). Compared to the cell body, CLASP1 siRNA promoted fast and long-lived MT growth within EC branches, independent of ECM composition, although the effects on growth lifetime were greatest on the fibronectin ECM (collagen GS=12.1 µm/min; GL=7.6 s; fibronectin GS=13.4 µm/min; GL=10.6 s; Fig. 4B-E). Evaluation of color-coded MT growth track overlays in CLASP1-depleted cells cultured on high-density fibronectin confirmed that ECs treated with CLASP1 siRNA displayed increased numbers of fast and long-lived MT growth tracks within branches as compared to the cell body (blue tracks, Fig. 4F). The finding that either siRNA-mediated depletion of CLASP1 or overexpression of GFP-CLASP1 caused a regional increase in MT growth lifetimes was unexpected, but highlights that CLASP1-mediated regional control of MT growth lifetimes is sensitive to both ECM composition and to modulation of CLASP1 expression.

To determine how CLASP1-mediated regulation of MT growth dynamics contributes to EC branching morphology, we plotted MT growth speed versus branch number or versus branch length (Fig. 4G). The results of these comparisons revealed that increased MT growth speeds displayed a negative linear correlation with increased EC branched number and displayed a positive linear correlation with EC branch length, both on high-density collagen-I and fibronectin ECMs (Fig. 4G). Together, these data suggest that CLASP1 promotes slow MT growth within EC branches and that CLASP1-induced slow MT growth promotes increased branch number while inhibiting overall branch length.

### CLASP1 promotes persistent and directional EC migration

**Fig. 5.**
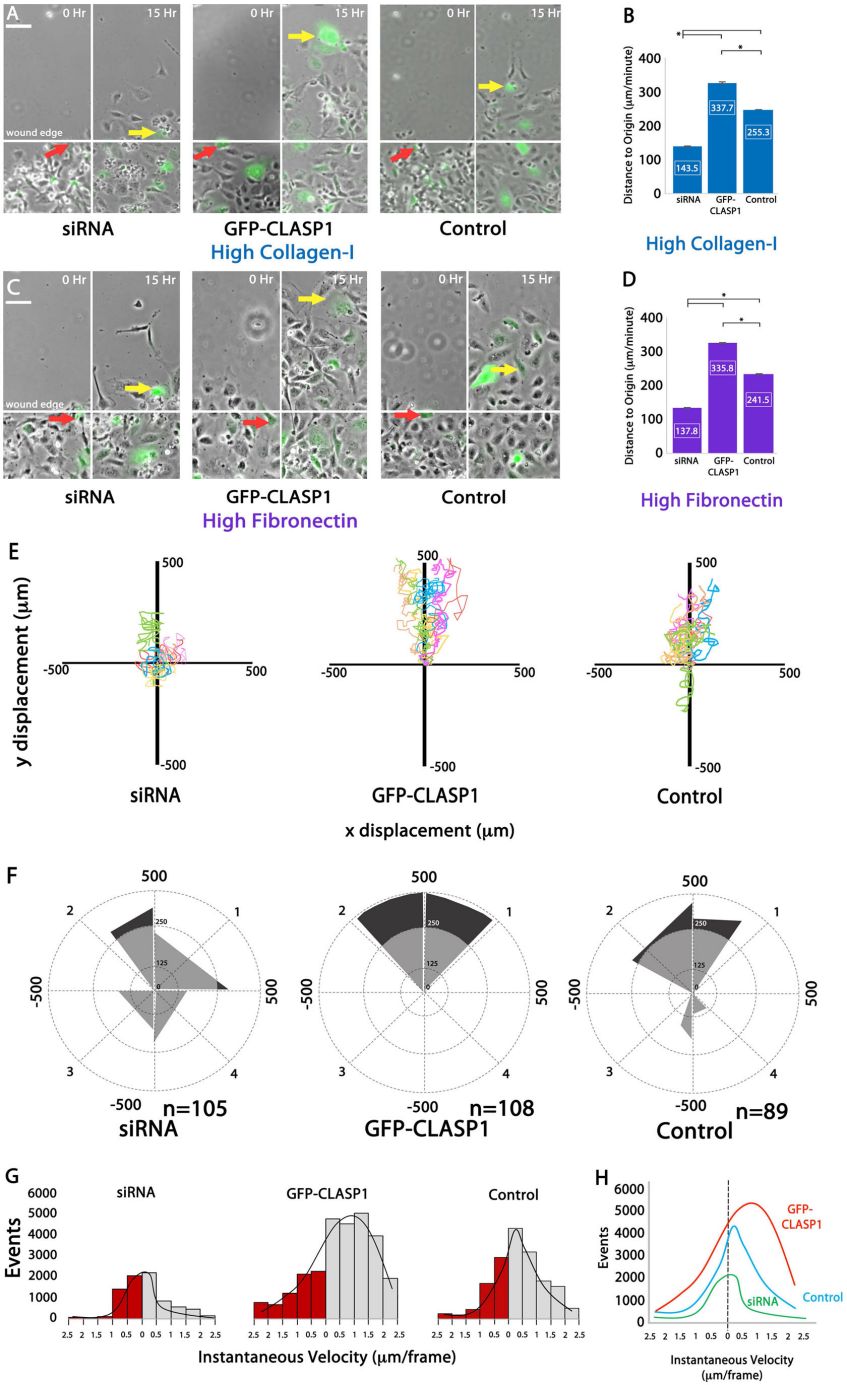
**CLASP1 promotes persistent and directional EC migration.** (A,C) Representative images of EC wound-edge migration on a high-density collagen-I ECM (A) or a high-fibronectin ECM (C). Red arrows represent GFP-labeled, wound-edge EC location at 0 h, and yellow arrows represent the final location of the GFP-labeled wound-edge EC at 15 h. Scale bars: 100 µm. (B,D) Mean migration distance measurements for ECs cultured on high-density collagen-I ECMs (B) or cultured on high-density fibronectin ECMs (D). (E) Migration displacement graphs of representative ECs for over a 15-h time period. (F) Rose plot diagrams of cell migration distance and direction. Quadrants 1 and 2 represent directionally positive movement into the wound edge, while quadrants 3 and 4 represent movement away from the wound edge. Black shading depicts migration events that were greater than or equal to a migratory distance of 250 µm, while gray shading depicts migratory events that were less than 250 µm. (G) Histogram showing instantaneous migration velocities binned into 0.5 µm intervals and plotted against the number of events for each interval. Bars in gray represent directionally positive movement into the wound edge. Bars in red represent movement away from the wound edge. (H) Distribution curve of instantaneous velocities for the binned intervals shown in G. *P<0.001; ANOVA. Error bars indicate ±s.e.m.

To identify the effects of CLASP1 and ECM composition on EC migration, we performed wound-edge migration assays on either high-density collagen-I or high-density fibronectin ECMs. Visual analysis demonstrated that EC directional migration was inhibited in CLASP1-depleted cells on both collagen-I and fibronectin ECMs (Fig. 5A,C). To quantitate this observation, EC migration from the starting position at the wound edge (t=0 h), to the final position (t=15 h) was measured (termed ‘distance to origin’). This analysis revealed that, compared to control ECs, CLASP1 siRNA significantly inhibited EC migration (collagen-I=44% reduction; fibronectin=43% reduction), while expression of GFP-CLASP1 increased EC migration distance to origin (collagen-I=132% increase; fibronectin=139% increase; Fig. 5B,D). These findings suggest that CLASP1 promotes directional EC migration on both collagen-I and fibronectin ECMs.

To evaluate directional persistence, EC migration displacement analysis was conducted (Fig. 5E). This analysis revealed that the most persistent directional migration into the wound occurred in ECs expressing GFP-CLASP1 (Fig. 5E). Additionally, rose plot analysis of migration persistence revealed that GFP-CLASP1 expressing ECs displayed a dominant forward movement that was restrained to quadrants 1 and 2 of the rose diagram, corresponding to migration persistence in excess of 250 µm into the wound (Fig. 5F).

Measurements of migration distance to origin and directional persistence are both composed of the sum of a variable number, and variable distance, of instantaneous movements. Thus, we sought to identify the contribution of individual migration measurements to the overall migration distance and persistence (represented in Fig. 5A-F). To do this, measured instantaneous migration velocities were binned into groups representing 0.5 µm/frame intervals (1 frame=10 min; total migration time=15 h) and were plotted as histograms with trendline distributions fit to each instantaneous velocity graph (Fig. 5G,H). Additionally, the number of movements were quantified and compared with respect to wound-edge migration directionality as either backward or forward (red versus gray bars in Fig. 5G; see also Table S1).

This analysis revealed a single-peak trendline for each experimental condition. In control ECs, the number of forward and backward movements were similar, with slightly more movements in the forward direction (control backward=12,192; control forward=13,804; control peak velocity=0.32 um/frame; total migration time=15 h). In CLASP1 depletion, where the overall migration distance to origin and persistence was inhibited (see Fig. 5B,D-F), ECs exhibited an increased number of backward movements relative to forward movements, and the total number of forward and backward movements was vastly reduced compared to control (siRNA backward=4152; siRNA forward=2199). Additionally, the peak instantaneous velocity in the siRNA group was halved compared to control (siRNA velocity=0.17 µm/frame; Table S1). In the GFP-CLASP1 group, where migration distance to origin and persistence was significantly increased (see Fig. 5B,D-F), the peak instantaneous velocity was measured at 0.89 µm/frame, nearly three times greater than the control peak instantaneous velocity, and more than five times greater than the CLASP1 siRNA peak instantaneous velocity (Fig. 5G,H). Additionally, in GFP-CLASP1 overexpressing ECs, the number of backward movements was reduced and the number of forward movements was increased compared to all other groups (GFP-CLASP1 backward=11,369; GFP-CLASP1 forward=16,079; Table S1). Together these data suggest that CLASP1 drives persistent and directional wound-edge EC migration by promoting both an increased number and an increased velocity of movements in the direction of the wound.

## DISCUSSION

The ECM manifests unique combinations of proteins, fibers and ligands that provide a structural platform for cell engagement, morphogenesis, and migration (Cukierman et al., 2002; Ingber, 2002; Rhodes and Simons, 2007; Bozzuto et al., 2010; Hynes and Yamada, 2013; Jiang et al., 2016). It has been shown that in response to ECM compliance and dimensionality mechanosensing, MT dynamics change in ways that contribute to the regulation of EC branching morphogenesis (Myers et al., 2011), suggesting that physical interactions between cells and the ECM regulate intracellular signaling mechanisms that target the regulation of MT growth. Various cell types are known to modulate the architecture of the cytoskeleton and thereby adapt their morphology in response to the density and composition of the ECM (Loftis et al., 2003; Beningo et al., 2004; Wantanabe et al., 2005; Kikuchi and Takahashi, 2008; Wolf and Friedl, 2011). Similarly, ECs have been shown to modulate their branching morphology through mechanosensitive responses that also require adaptive regulation of the MT cytoskeleton (Kaverina et al., 1999; Wadsworth, 1999; Myers et al., 2011; for review see Kaverina and Straube, 2011; Carey et al., 2015). Here, we set out to test the hypothesis that the composition and density of the ECM drives the regulation of MT growth dynamics by targeting the MT stabilizing protein, CLASP1, in order to control EC branching and directional migration.

Our results consist of four main findings that identify the contribution of ECM-induced signaling to CLASP1-mediated regulation of EC MT dynamics. First, we found that ECM density regulates MT growth dynamics in a CLASP1-dependent manner, whereby high-density ECMs promote long-lived MT growth, and this effect is enhanced by CLASP1-knockdown and is attenuated by CLASP1 overexpression. Second, we identified that MT growth dynamics are adaptive to changes in ECM composition, such that the fastest and longest-lived MT growth tracks were measured in ECs cultured on type-I collagen ECMs. Third, we showed that CLASP1 modulates MT growth dynamics differently within cell branches as compared to the cell body. Fourth, we determined that CLASP1 promotes persistent and directional EC wound-edge migration.

Our finding that CLASP1 regulates MT growth dynamics in response to ECM density is in support of previous investigations showing that MT dynamics are indeed sensitive to physical signals from the ECM (Lo et al., 2000; Rhee et al., 2007; Myers et al., 2011). Our results also highlight that the sensitivity of CLASP1-mediated regulation of MT dynamics is heightened on high-density ECMs, suggesting that the effects of CLASP1 on MT dynamics can be tuned to complement the composition of the ECM. For example, in control ECs on uncoated glass coverslips (in the absence of ECM ligand), MT growth dynamics were similar to those measured in control ECs on low-density collagen-I or on low-density fibronectin ECMs. However, comparison of either low-density ECM substrate following manipulation of CLASP1 resulted in significantly altered MT dynamics when compared to glass ECMs. Importantly, comparison of ECM density revealed that CLASP1 displayed its most prominent effects on MT growth lifetimes, with fewer effects on MT growth speeds. Our studies also revealed that the effects of CLASP1 on low-density ECMs were dampened compared to high-density ECMs, where CLASP1 altered both MT growth speed and growth lifetime. It was at first surprising to see that CLASP1 knockdown induced the most dramatic increase in MT growth lifetimes, while overexpression of CLASP1 only reduced MT growth lifetimes to control levels. Experimental analysis revealed that ECM composition alone did not drastically alter endogenous CLASP1 expression, supporting our conclusion that ECM-induced changes in MT growth dynamics were CLASP1-dependent. Additionally, these data suggest that endogenous levels of CLASP1 are maximally efficient at reducing MT growth lifetime; or alternatively, that the measured lifetimes may represent a lower limit for the CLASP1-meditated regulation of growth lifetime.

It is important to note that there are two mammalian CLASP homologs, CLASP1 and CLASP2, and both have been shown to have a role in modulating MT dynamics by reducing microtubule catastrophe events (Mimori-Kiyosue et al., 2005). Here, we chose to focus our EC investigations on the CLASP1 protein, because a previous study revealed that CLASP1 is robustly expressed in vascular tissues, including heart and lung, among others, while CLASP2 is primarily expressed in brain tissue, with limited expression in other tissue types (Akhmanova et al., 2001). Using measurements of individual MT growth parameters, our data show that high-density type-I collagen ECMs induced the fastest and longest-lived MT growth tracks without modulating CLASP1 protein levels. Meanwhile, data from CLASP1 knockdown experiments show an induction of fast and long-lived MT growth on both collagen and fibronectin ECMs, suggesting that it is not only the amount of CLASP1 protein expressed in ECs, but that there is an important regulatory component that must target CLASP1 for activation or inhibition. Indeed, previous investigations have characterized Rac1 and GSK3B intracellular signaling cascades that target CLASP-mediated regulation of MT stability (Wittmann and Waterman-Storer, 2005; Kumar et al., 2009; Trogden and Rogers, 2015). Our findings do not exclude the possibility that EC engagement of collagen-I versus fibronectin ECM composition may function downstream of a Rac1 signaling pathway, or possibly through the actions of other MAPs that stimulate MT assembly or disassembly. Recent evidence implicates CLASP1 as playing a more direct role in linking MT dynamics to focal adhesion dynamics (Stehbens et al., 2014; Lim et al., 2016). Thus, it is possible that CLASP1 localized within, or adjacent to, focal adhesion complexes (for example, within branched protrusions) may be sufficient to locally modify MT dynamics using Rac1-mediated or Rac1-independent signaling mechanisms.

The physical composition of the ECM plays a critical role in cell branching morphology (Ingber, 2002; Fischer et al., 2009; Doyle et al., 2015). We therefore hypothesized that the CLASP1-mediated effects on MT growth dynamics measured in the whole cell would reveal regional specificity within branched protrusions. Experimental evaluation of regional MT data within EC branched protrusions confirmed that slow and short-lived MT growth is conducive to the formation of new branched protrusions, while fast and long-lived MT growth is conducive to branch elongation. Of particular interest was the finding that growth lifetimes were the longest-lived within branches on fibronectin ECMs (see Fig. 4E). This data was initially counter-intuitive because our measurements of whole-cell MT growth dynamics had suggested that high-density collagen-I ECMs had the longest MT growth lifetimes. Further analysis of MT growth speeds and lifetimes on an individual, cell-by-cell basis (see Figs S1 and S2) revealed that this outcome is specific to MT growth lifetimes, a measurement that is inherently bound by the unique spatial length of each branched protrusion. Thus, because fibronectin branches were significantly longer than collagen-I branches (see Fig. 3D), MTs within fibronectin branches were provided increased spatial capacity to extend their growth lifetimes. Nevertheless, our findings show that CLASP1 knockdown results in significantly faster and longer-lived MT growth specifically within EC branches, and thereby identify CLASP1 as a critical regulator of MT dynamics within EC branches.

EC branching morphology is known to be a driving force underlying the directional persistence of EC migration (Kearney, 2004; Larsen, 2006; Fischer et al., 2009; Hsu et al., 2014; Elliott et al., 2015). Additionally, cell branching morphology and migration are known to be critically dependent upon the physical composition of the ECM, which can act to stimulate or repress branched protrusions through modulation of cytoskeletal architecture (Georges and Janmey, 1985; Ingber, 2002, 2006; Ghosh et al., 2008; Doyle et al., 2009; Myers et al., 2011; Doyle et al., 2015). In this way, the ECM dictates both the availability for cell-ECM interactions, as well as the propensity for physical interactions and force generation needed to advance the cell relative to the ECM (Ingber, 1997; Sheetz et al., 1998; Galbraith et al., 2002; Peyton and Putnam, 2005; Maruthamuthu et al., 2010; Plotnikov et al., 2012; Wolf et al., 2013; Thievessen et al., 2015). Our data support these previous investigations, and further identify CLASP1 as key regulator of wound-edge EC migration in response to the composition and density of the ECM. We interpret these experimental migration outcomes to be the sum result of CLASP1-mediated regional regulation of MT growth dynamics that contribute to modulating both the number and length of branched protrusions. Specifically, within branched protrusions, CLASP1 promotion of slow MT growth translated into an increase in the number of short branches and an overall increase in migration persistence and distance to origin. Our analysis further revealed that CLASP1-driven increases in directional migration resulted from an increased number and greater step size in the forward direction, as compared to the backward direction. Interestingly, our examination of instantaneous EC movements highlights that ECs under all experimental conditions of CLASP1 were still able to move forward into the wound. Importantly, we found that only in the CLASP1 overexpression group were both the sum number of movements and the velocity of those movements increased in the forward direction. Thus, the overall directional persistence of EC wound-edge migration is guided by branched protrusions that are numerous and short, are promoted via cell engagement with high-density 2D fibronectin and collagen-I ECMs, and that are driven by CLASP1-induced slow MT growth. Future investigations will prove essential to determine the relationship between CLASP1 regulation of MT growth dynamics in 3D ECMs, where ECM engagement by branched protrusions and EC migration are likely to involve additional challenges for cytoskeletal reorganization.

## MATERIALS AND METHODS

### Cell culture

HUVECs (ECs) were cultured and maintained in EBM medium supplemented with EGM-MV Single Quots (Lonza, Walkersville, MD, USA) and penicillin/streptomycin (RPI) at 37°C with 5% CO_2_. Transfection of siRNA and/or cDNA was performed using a Nucleofector Device with solution kit V (Lonza), setting A-034. ECs were cultured at 50,000 cells/coverslip for live-cell imaging and branching morphogenesis, or 500,000 cells/coverslip for wound-edge migration on either uncoated glass, 2D collagen-I, or 2D fibronectin-coated glass coverslips (see 2D substrate below). Experiments were performed 8-12 h after transfection to allow time for protein expression and/or siRNA knockdown. Before live-cell imaging, the exposed cell surface was supplemented with 25 µm Hepes, pH 7.2, diluted with 1× PBS (Lonza), to maintain physiological pH.

### 2D substrates

Working on ice, 9 mg/ml high purity rat-tail collagen-I (Corning, NY, USA) was diluted to 300 µg/ml, and 150 µl was combined with 48 µl 10× MEM, 270 µl dH_2_0, and 36 µl NaCO_3_ to create a 90 µg/ml concentration (high-density). Quantities were adjusted (150 µl 300 µg/ml collagen-I, 96 µl 10× MEM, 690 µl dH_2_0, and 72 µl NaCO_3_) to create a 45 µg/ml concentration (low-density). ‘Squeaky clean’ 22 mm×22 mm No. 1.5 coverslips (Corning) were prepared as previously described (Myers et al., 2011). In brief, coverslips were incubated in 0.5% 3-aminopropyl-trimethyoxysilane diluted in water (10 min on a stir plate), followed by six times 5-min water washes. Coverslips were dried at 37°C, cooled to room temperature, and then immersed in 95% ethanol solution and stored. Each coverslip was flame sterilized before coating with 250 µl collagen-I. Coverslips were then incubated at 4°C overnight in a polystyrene 30 mm petri dish (VWR, Radnor, PA, USA). Similarly, 1 mg/ml fibronectin (EMD) was diluted with 1× PBS (Lonza) to a 10 µg/ml (low) or 100 µg/ml (high) concentration and incubated at 37°C overnight. After overnight incubation, coverslips were washed three times with 1× PBS (Lonza).

### Expression constructs

cDNAs, labeled with GFP (GFP-CLASP1) or mApple (mApple-EB3), were generated by the Michael Davidson lab (Florida State University, USA) and provided by the Clare Waterman lab (NIH/NHLBI; Bethesda, MD USA). All cDNA used for transfection was prepared using the Plasmid Maxi kit (Qiagen). siRNAs used for knockdown (CLASP1-siRNA) and NT control (GENOME^®^ non-target siRNA) transfection experiments were purchased from Dharmacon Inc. (Lafayette, CO, USA) and re-suspended in RNAse free dH_2_0. CLASP1-siRNA custom sequence: 5′ GGGUAUUACUGACGAGCUUU 3′. NT control sequence: 5′ GAACAUUGCUGGACGUCCUUU 3′.

### Microscopy and image analysis

Microscopic imaging was performed on a spinning disk (Yokagawa CSU-X1; Andor Technology, Belfast, UK) confocal microscope using a 60×1.4 NA oil immersion objective lens on a TiE microscope equipped with Perfect Focus System (Nikon) equipped with an electronic shutter (Sutter Instrument, Novato, CA, USA) for transmitted illumination, a linear encoded X and Y, motorized stage [Applied Scientific Instrumentation (ASI), Eugene, CA, USA], and a multi-bandpass dichromatic mirror (Semrock, Rochester, NY, USA) and bandpass filters (Chroma Technology Corp., Bellows Falls, VT, USA) in an electronic filterwheel for selection of GFP or RFP emission. 488- and 561-nm laser illumination was provided by a high-powered (50 mW 488- and 561-nm) monolithic laser combiner module (MLC 400B; Agilent Technologies, Wilmington, DE, USA) with electronic shutters and directed to a fiber-coupled output port with an Acousto optic tunable filter and to the confocal scan-head via a singlemode polarization-maintaining fiber coupled delivery system (Agilent Technologies). Images were acquired using a Clara cooled CCD camera (Andor) operated in the 14-bit mode for 2 min at 2-s image intervals using a 300-400 ms exposure time. Microscope system automation was controlled with NIS Elements software (Nikon). After acquisition, image processing involved optimization of image brightness and contrast using the acquisition software package and were applied uniformly to all images. Additional microscopy image manipulations and measurements are described in detail in the appropriate Materials and Methods subsections.

### MT dynamics analysis

MT dynamics were analyzed from fluorescently-labeled EB3 (mApple-EB3) movies using plusTipTracker software, a MATLAB-based, open-source software package that combines automated detection, tracking, analysis, and visualization tools for movies of fluorescently labeled MT plus end binding proteins as previously described (Jaqaman et al., 2008; Applegate et al., 2011; Myers et al., 2011; Braun et al., 2016; D'Angelo et al., 2017). All MT growth parameter tracking and EC branching experiments were performed four times.

### Determination of image quality

Detection, tracking, and post-processing analysis were performed on the first 30 frames only for each movie, as photobleaching was a limiting feature in some conditions and movie length was standardized for tracking comparison. The quality of the movies was assessed by examining comet detection performance; movies were discarded from further analysis if EB3 expression was too high and led to too many false positives, or if focus drift or photobleaching led to a high standard deviation in mean comet number per frame over the course of the movie.

### Tracking parameters

Tracking control parameters were optimized based on a parameter sweep using the plusTipParamSweepGUI tool of plusTipTracker (Applegate et al., 2011) and verified by visual inspection of track overlays on movies. The same parameter set was used for all movies in the dataset, as previously described (Myers et al., 2011). In brief, tracking occurs in two steps: frame-to-frame linking of EB3 comets into growth sub-tracks, and the linking of collinear, sequential growth sub-tracks into compound tracks. The cost of joining two candidate growth sub-tracks into a compound track is calculated from three spatial parameters and one temporal parameter. After calculating the cost of linking all pairs of candidate growth tracks, the links are chosen by minimizing the global cost, which is achieved by solving the linear assignment problem (Jaqaman et al., 2008). The same parameter set was used for all movies in the dataset: maximum gap length, 12 frames; minimum track length, 3 frames; search radius range, 5–10 pixels; maximum forward angle, 25°; maximum backward angle, 8°; maximum shrinkage factor, 1.0; fluctuation radius, 2 pixels. For this study, only growth excursions were of interest, so MT shrinkage or pause events were not analyzed. However, sub-track linking was still performed to correct for the many occurrences when comets cross over one another or disappear momentarily from the field of view by focal drift, which breaks the trajectories prematurely. Measurements of MT growth speeds were recorded as the number of microns per minute as determined by the camera pixel dimensions and the time intervals for image acquisition (2 s intervals). Measurements of MT growth lifetimes were similarly calculated using the time intervals for image acquisition and were defined as the length of time that an EB3 comet was detected and linked within a single compound track by plusTipTracker software. To reduce false positives, each comet was required to be detected using the specified parameters for a minimum of 6 s (3 imaging frames) in order for it to be linked in to a compound track.

### Region of interest (ROI) selection

Binary masks of whole cells and/or individual branches were generated in two steps using plusTipTracker's sub-ROI selection tool. First, whole cell and branch masks were manually selected based on the whole cell outlines and branch definitions. The subtraction of branch pixels was accomplished by choosing the all-branches mask generated in the first step as an exclusion mask in the second. Regional areas were calculated by summing the area of the mask and converting to square micrometers. The set of all MT growth excursions that spent a minimum of 6 s (3 imaging frames) within an ROI were extracted and stored within a subproject of the original cell. ROIs divide cells into sub-regions that are smaller than the whole cell. This results in some of the whole cell MT growth excursions being excluded from regional analysis, because growth excursions must exist within either the cell body ROI, or the cell branch ROI, for the minimum time of 6 s. Due to the creation of spatial boundaries that are smaller than the whole cell, regional analysis can result in reduced MT growth lifetimes, but does not typically affect MT growth speeds. Because of this, regional MT growth dynamics data were compared separately from whole-cell MT growth dynamics data in all cases.

### MT growth sub-track subpopulation analysis

Tracks from within ROIs from all movies in the dataset were pooled using the plusTipPoolGroupData function to find the mean MT growth speed and growth lifetime. These values were used to conduct quadrant plot analysis (Applegate et al., 2011), which was performed as separate batch processes for whole cell, cell body, and branches, thus defining for each of the groups the total number of tracks in each of the four subpopulations: slow and short-lived, slow and long-lived, fast and short-lived, and fast and long-lived. The threshold used for characterizing MT growth tracks as slow versus fast, and short-lived versus long-lived, was the mean value from all data sets for MT growth speed (slow versus fast) and MT growth lifetime (short-lived versus long-lived). The relative proportions of these four subpopulations were used to generate MT growth track overlays for visual comparison.

### MT tracking statistical analysis

The MT tracks visualized within each individual ECs were pooled and taken collectively as one event. MT growth speeds and lifetimes were firstly divided into respective CLASP1 expression profiles (siRNA depletion, GFP-CLASP1 overexpression, and Control) and averaged. For MT growth track and branching morphogenesis experiments, a minimum of 10 cells was examined for each condition. For migration experiments, a minimum of 89 ECs were examined and experiments were performed in triplicate and are expressed as mean±s.e.m. generated in Excel. Using GraphPad Prism software, a one-way ANOVA test with post hoc Tukey's analysis was performed to determine statistical significance. For MT tracking, the α was set to 0.05 and for branching morphogenesis and wound-edge migration assays the α was set to <0.001 for statistical significance (*n*=number of cells or *n*=number of branches). For all statistics reported, the sample number (*n*) used for each sample comparison was determined to equate to a power value greater than or equal to 0.8 using a two-sided analysis for means.

### Cell migration assay

Wound-edge migration assays were performed on 22-mm round No. 1.5 coverslips prepared with high-density collagen-I or high-density fibronectin coating (as described above), but with the following modifications. Before plating the cells, the coated coverslips were dried using an aspirator. 500,000 transfected ECs were then cultured in 100 µL of EBM medium and were allowed to adhere to the coverslip and form a confluent monolayer. The monolayer was then rinsed two times in EBM medium to remove unattached cells and the monolayer was then scraped with a razor blade to generate a wound edge. The cells were then allowed to recover in a 37°C incubator for 2 h before being mounted in a custom-built multi-position stage insert. Phase-contrast images were acquired at 10-min intervals for 15 h on the microscope using a 10×0.45 NA phase objective and a 0.52 NA LWD condenser using the NIS Elements ND acquisition software module. All time-lapse wound-edge migration assays were performed a minimum of three times.

### Quantification of cell branching and migration

For analysis of cell branching, branches were defined as protrusions that extended from the cell >10 µm in length and were longer than they were wide. Branches were counted only if they met this criterion. ‘Branch origin’ and ‘branch length’ were measured as previously described (Myers et al., 2011). Cell migration was performed using blinded analysis in duplicate, to ensure reproducibility, and was quantified by hand-tracking the nucleolus in successive images from a time-lapse phase-contrast image series using the MTrackJ tracking Plugin of ImageJ (NIH). Migratory displacement measurements were obtained through subtraction of each x and y coordinate from its predecessor. Individual displacement values were then binned into one of four categories, based on their overall directionality (Quadrant I, II, III, IV). Each displacement value was further qualified by distance (<250 µm or >250 µm). The total percentage of values that fell within each quadrant was binned and graphically represented in angular rose plot diagrams, where light gray represents values <250 µm and dark gray represents values >250 µm. Distance to origin was obtained using the ‘straight line tool’ in ImageJ. Instantaneous velocities were binned into 10 µm intervals and plotted into a histogram in Excel; a distribution curve was fitted to each histogram. Statistical analysis was performed using the Analyze-It plug-in for Microsoft Excel (Analyze-It Software, Ltd.; https://analyse-it.com/), and branching and migration data were compared using a two-tailed student *t*-test, with α set to 0.001 for statistical significance.

### Immunoblotting

Cytoplasmic extracts from transfected ECs were isolated in NP40 lysis buffer (50 mM Tris, pH 8.0, 150 mM NaCl, 0.5% sodium deoxycholate, 1% nonidet P-40, 1 M NaFl, and 100X Halt Protease Inhibitor Cocktail) and manually collected by scraping. After 10-min lysis incubation on ice, cell nuclei were removed via centrifugation. Protein concentrations from supernatants were quantified by Bradford assay. 15 µg of protein from each sample was mixed with Laemmli sample buffer and separated by SDS-PAGE. After electrophoresis, proteins were electrotransfered to a nitrocellulose membrane. For protein detection, membranes were blocked for 30 min at room temperature with 5% fat-free milk in TBST buffer (20 mM Tris, pH 7.6, 137 mM NaCl_2_ and 0.1% Tween-20) and incubated for one hour at room temperature with rabbit pAb GFP (1:3000; Ab290), rabbit mAb CLASP1 (1:3000; Ab108620), and/or mouse mAb GAPDH (1:3000; Ab9484). After primary antibody incubation, blots were washed one time with TBST for 15 min and then two times for 5 min each and incubated with the appropriate HRP-conjugated secondary antibodies (1:25,000; Jackson Immunoresearch Laboratories, Inc.) for 30 min. Blots were washed one time for 5 min and then four times for 15 min each with TBST and protein bands were visualized using ECL detection system (SuperSignal West Pico or Dura Chemiluminescent Substrate; Thermo Fisher Scientific). For all quantitation of protein densitometry, local background subtraction was first performed around bands of interest in accordance with Gassmann et al. (2009). Next, experimental CLASP1 and/or GFP-CLASP1 bands were normalized to respective GAPDH loading controls, followed by measurements of the normalized digital images of immunoblot bands using with ImageJ software (NIH). To calculate the total amount of CLASP1 protein for each experimental condition, densitometry measurements for the GFP-CLASP1 band were added to the CLASP1 band for all experimental conditions. Quantitation of the GFP-CLASP1 overexpression condition (Fig. 2E-H) were subsequently labeled ‘Total CLASP1’ in order to distinguish this measurement from the GFP-CLASP1 band alone. Quantification of CLASP1 densitometry and reported fold changes (Fig. 2E-H) and percentage changes (‘Results’ section) were calculated relative to the ‘Control’ sample (a.k.a. were normalized to control) for individual ECM composition and density conditions, and were not compared between ECM conditions. Endogenous CLASP1 densitometry measurements (Fig. 2I) were obtained and compared between ECM conditions in a separate western blot (data not shown).
